# Preserved Antigen-Specific Immune Response in Patients with Multiple Sclerosis Responding to IFNβ-Therapy

**DOI:** 10.1371/journal.pone.0078532

**Published:** 2013-11-05

**Authors:** Matthias Mehling, Stefanie Fritz, Patricia Hafner, Dominik Eichin, Tomomi Yonekawa, Thomas Klimkait, Raija L. P. Lindberg, Ludwig Kappos, Christoph Hess

**Affiliations:** 1 Immunobiology Laboratory, Department of Biomedicine and Medical Outpatient Department, University Hospital Basel, Basel, Switzerland; 2 Department of Neurology and Clinical Neuroimmunology Laboratory/Department of Biomedicine, University Hospital Basel, Basel, Switzerland; 3 Medical Image Analysis Center, University Hospital Basel, Basel, Switzerland; 4 Institute of Medical Microbiology, Department of Biomedicine, University of Basel, Basel, Switzerland; University Hospital La Paz, Spain

## Abstract

**Background:**

Interferon-beta (IFNβ) regulates the expression of a complex set of pro- as well as anti-inflammatory genes. In cohorts of MS patients unstratified for therapeutic response to IFNβ, normal vaccine-specific immune responses have been observed. Data capturing antigen-specific immune responses in cohorts of subjects defined by response to IFNβ-therapy are not available.

**Objective:**

To assess antigen-specific immune responses in a cohort of MS patients responding clinically and radiologically to IFNβ.

**Methods:**

In 26 MS patients, clinical and MRI disease activity were assessed before and under treatment with IFNβ. Humoral and cellular immune response to influenza vaccine was prospectively characterized in these individuals, and 33 healthy controls by influenza-specific Enzyme-Linked Immunosorbent Assay (ELISA) and Enzyme Linked Immuno Spot Technique (ELISPOT).

**Results:**

Related to pre-treatment disease activity, IFNβ reduced clinical and radiological MS disease-activity. Following influenza vaccination, frequencies of influenza-specific T cells and concentrations of anti-influenza A and B IgM and IgG increased comparably in MS-patients and in healthy controls.

**Conclusions:**

By showing in a cohort of MS-patients responding to IFNβ vaccine-specific immune responses comparable to controls, this study indicates that antigen-specific immune responses can be preserved under successful IFNβ-therapy.

## Introduction

IFNβ, as all type I interferons (IFNα, IFNβ, IFNe, IFNk, IFNx, and IFNω), binds to the IFNα receptor (IFNAR) [1], resulting in phosphorylation of *signal transducer and activator of transcription* (STAT) complexes that regulate the expression of a complex set of pro- as well as anti-inflammatory genes [2]. In patients with relapsing MS, IFNβ suppresses in a portion of patients clinical and subclinical inflammatory autoimmunity via a variety of (postulated) mechanisms (reduced T cell mediated inflammation, altered function of antigen-presenting and other immune cells, stabilization of the blood-brain barrier) [3]–[10], while no signs of a general immunosuppressive effect have been noted. Also, non-suppressed vaccine-induced inhibition of hemagglutination suggested some degree of selectivity of IFNβ in suppressing autoimmune inflammation [11], [12]. However, these studies were done in cohorts of patients that were not defined with regard to their response to IFNβ-therapy. Therefore, potential subclinical immuno-inhibitory effects of IFNβ in subjects responding to IFNβ-therapy may have been concealed. In search of a potential (subclinical) immuno-inhibitory effect of IFNβ we here prospectively monitored humoral and cellular vaccine-specific immunity in a cohort of patients with MS defined by clinical and radiological response to IFNβ-treatment as well as in healthy controls.

## Patients and Methods

### Study subjects and procedures

An open-label, observational, combined retrospective and prospective study was performed aiming (i) to assess in patients with MS the clinical and MRI response to initiation of IFNβ-treatment (retrospective part) and (ii) to compare the adaptive immune response induced by influenza-vaccination in the same cohort of patients with MS under established IFNβ-therapy, and in healthy controls (HC) (prospective part). The institutional review board of Basel approved the study. After written informed consent, blood samples from study subjects were obtained before and 7, 14 and 28 days after seasonal influenza-vaccination with Mutagrip® (Sanofi Pasteur SA, Lyon). The prospective part of the trial was conducted during the influenza-vaccination periods 2008/2009 and 2009/2010. Inclusion criteria for patients at the time of recruitment into the prospective part of the study were definite relapsing MS, treatment with IFNβ, and age ≥18 and ≤65 years. Inclusion criteria for healthy controls (prospective part of the study) were absence of chronic disease, and age ≥18 and ≤65 years. Exclusion criteria for patients and controls were known hypersensitivity to the vaccine under investigation, fever at time of planned vaccination, influenza vaccination <180 days before recruitment into the study, treatment with immunoglobulins or exogenous blood products within 90 days before recruitment into the study, simultaneous medication with steroids or immune-therapy other than IFNβ and pregnancy. The institutional review board of both cantons of Basel approved the study. Retrospectively, the annualized relapse rate and the number of new T2-lesions/year in MRI were assessed in the study participants with MS before and after initiation of IFNβ-treatment, *excluding relapses and new T2 lesions 3 months before and after initiation of IFNβ-treatment*. MRI data were analysed by a single neuroradiologist –which was blinded for the immunologic outcomes of our study– to reduce inter-rater variability. For the prospective assessment of the adaptive immune response induced by influenza-vaccination, blood samples from study subjects were obtained before and 7, 14 and 28 days after seasonal influenza-vaccination with Mutagrip® (Sanofi Pasteur SA, Lyon). Study participants were interviewed and examined before and 28 days after influenza-vaccination. In patients with MS, the expanded disability status scale (EDSS) score was assessed before and under treatment with IFNβ, including prospective assessments on day 0 and day 28 post vaccination. All study participants received a symptoms diary to document side effects of the vaccination, and flu-like symptoms. Results of influenza-vaccine induced immune responses in the influenza-vaccination periods 2008/2009 and 2009/2010 were tested for comparability, and only subsequently pooled for the final analysis.

### Enzyme linked immuno-spot assay

Enzyme linked immuno-spot (ELISpot) was done as described previously[13] with using Inflexal® (Berna Biotech, Kuesnacht, Switzerland) as source of antigen (year adjusted). In brief, ELISpot plates (MSIPS4510, Millipore AG, Volketswil, Switzerland) were coated with 2 µg/mL of anti-IFN-gamma mAb 1-D1K (Mabtech, Nacka Strand, Sweden) overnight. In each well 200.000 peripheral blood mononuclear cells (PBMC) were added in R10 (RPMI 1640 containing 10% heat inactivated Fetal Bovine Serum [FBS], 50 U/mL penicillin and 50 µg/mL Streptomycin [all from GIBCO™, LuBioScience GmbH, Luzern, Switzerland]) (final volume 130 µl/well). All measurements were performed in duplicates. Inflexal® (Berna Biotech, Kuesnacht, Switzerland) was used as source of antigen (year adjusted) at a final concentration of 14 µg/mL for each peptide, phytohemagglutinin (PHA) (1.8 µg/mL; REMEL, Oxoid AG, Basel, Switzerland) served as a positive control. Plates were incubated for 16 hours at 37°C with 5% CO_2_, washed with PBS (phosphate-buffered saline) and blocked with PBS 1% FBS. After washing, plates were incubated with 100 µl anti-IFN-gamma mAb (1∶200) coupled with alkaline phosphatase (7-B6-1-ALP, Mabtech) for 2 hours at room temperature. Spots were developed with HistoMark RED phosphatase system (KPL, Gaithersburg, Maryland, USA) and counted with the ELISpot Reader System (CSR01, AID GmbH, Strassberg, Germany) using the ELISpot 3.5 software (AID GmbH). 50 spot forming cells (SFC)/10^6^ PBMC were defined as cut-off for a positive antigen-specific response.

### Anti-influenza A and anti-influenza B enzyme-linked immunosorbent assay

Concentrations (given as virotech [VE] units/mL) of IgM and IgG anti-influenza A and anti-influenza B were determined in quadruplicates using a quantitative enzyme-linked immunosorbent assay (ELISA) according to the manufacturer (Genzyme Virotech, Ruesselsheim, Germany). As recommended by the manufacturer, seroprotection was defined as an anti-influenza A/B IgG-concentration of ≥10 VE/mL.

### Statistical analyses

Data were tested for normality with the Shapiro-Wilk test and Levene’s test was used to assess the equality of variances. Mann-Whitney test was performed in case of non-normality and/or differing variance among study-groups. Data with normal distribution were assessed by paired Student’s two-sided t-test. Fisher’s exact test was used for categorical analysis. Values of p< 0.05 were considered to be statistically significant.

## Results

### Study individuals, effects of IFNβ-therapy on MS, and tolerability of influenza vaccination

26 patients with MS and 33 healthy controls were recruited into the study. Characteristics of the study population are summarized in **Table 1** (upper part). In patients with MS, the annualized relapse rate decreased after initiation of treatment with IFNβ from 1.28 to 0.59 (p = 0.002). Likewise, the number of new T2-lesions/year decreased after initiation of IFNβ-therapy (before IFNβ-therapy: 1.8, under IFNβ-therapy: 0.6; p = 0.002) (**Table 1**, middle part). Importantly, all patients of our cohort hade experienced a reduction of the annualized relapse rate and in all patients in which MRI data were available had a reduction of the number of new T2-lesions/year. Following influenza-vaccination, rates of local injection site reactions were comparable in IFNβ-treated patients and in HC, while general symptoms occurred significantly more frequent in IFNβ-treated patients with MS (p = 0.0058) (perhaps more adequate p = 0.006 ?) (**Table 1**, lower part). Patients with MS and healthy controls did not differ in the frequency of influenza vaccination in the previous years.

**Table 1:** Study subject characteristics.

	healthy controls	MS IFNβ
	baseline characteristics	
N	33	26
median age (years) [range]	38 [19–46]	40 [29–49]
female/male	20/13	22/5
median disease duration (years) [range]	N.A.	3.9 [0.5–12.8]
median EDSS [range]	N.A.	2.5 [1.0–4.0]
median therapy duration (months) [range]	N.A.	44.1 [6–144]
	response to IFNβ-therapy	
annualized relapse rate before IFNβ-therapy	N.A.	1.28
annualized relapse rate under to IFNβ-therapy	N.A.	0.59
reduction of annualized relapse rate under IFNβ-therapy	N.A.	0.69 (p = 0.002)
new T2-lesions/year before IFNβ-therapy	N.A.	2.9
new T2-lesions/year under IFNβ-therapy	N.A.	0.8
reduction of new T2-lesions/year under IFNβ-therapy	N.A.	2.1 (p = 0.032)
flu-like symptoms after initiation of IFNβ-therapy	N.A.	69%
flu-like symptoms under established of IFNβ-therapy	N.A.	33%
IFNβ-preparation	N.A.	IFNβ-1a im OW: 9
		IFNβ-1a sc THW: 6
		IFNβ-1b sc EOD: 11
	tolerability of vaccine / incidence of influenza-like illness	
injection-site reactions day 0–3 post vaccination	21/33 (64%)	20/26 (77%)
general symptoms day 0–3 post vaccination	6/33 (18%)*	14/26 (54%)*
MS relapses	N.A.	3/26 (12%)
Incidence of influenza-like illness	4/33 (12%)	3/26 (12%)

Characteristics of the study population (upper part), clinical and subclinical response of patients with MS to IFNβ-therpay (middle part) and tolerability of influenza vaccination and incidence of influenza-like illnesses (lower part). Abbreviations: interferon-beta (IFNβ), IFNβv-treated patients with multiple sclerosis (MS IFNβ), not applicable (N.A.), Expanded Disability Status Scale (EDSS), intramuscular (im), subcutaneous (sc), once weekly (OW), three times per week (THW), every other day (EOD).

* indicates p<0.05.

### Humoral vaccine-specific immune response

**Figure 1:**
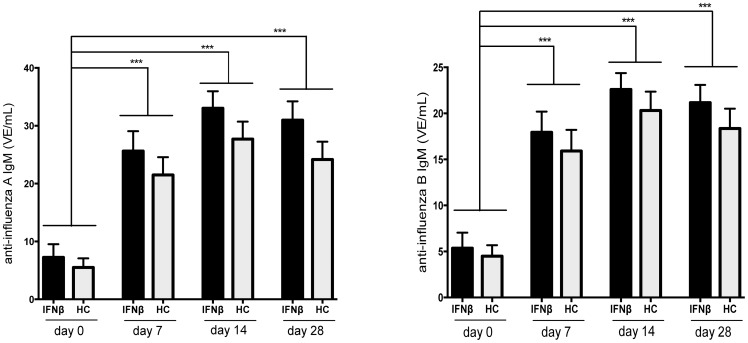
Anti-influena IgM-response after influenza-vaccination in IFNβ-treated patients and in healthy controls. The concentration of anti-influenza A (panel A) and anti-influenza B (panel B) IgM is shown as detected before (day 0) and at day 7, 14 and 28 after influenza vaccination in IFNβ-treated patients with MS (IFNβ) and healthy controls (HC) (mean + SEM). *** indicates p< 0.0001

Pre-vaccination levels of IgM directed against influenza A and B were comparably low in IFNβ-treated patients and in HC. Following vaccination, concentrations of influenza A- and B-specific IgM increased significantly and comparably in both study groups, and remained increased at comparable levels on day 28 post vaccination (**Fig. 1A**/**B**).

**Figure 2:**
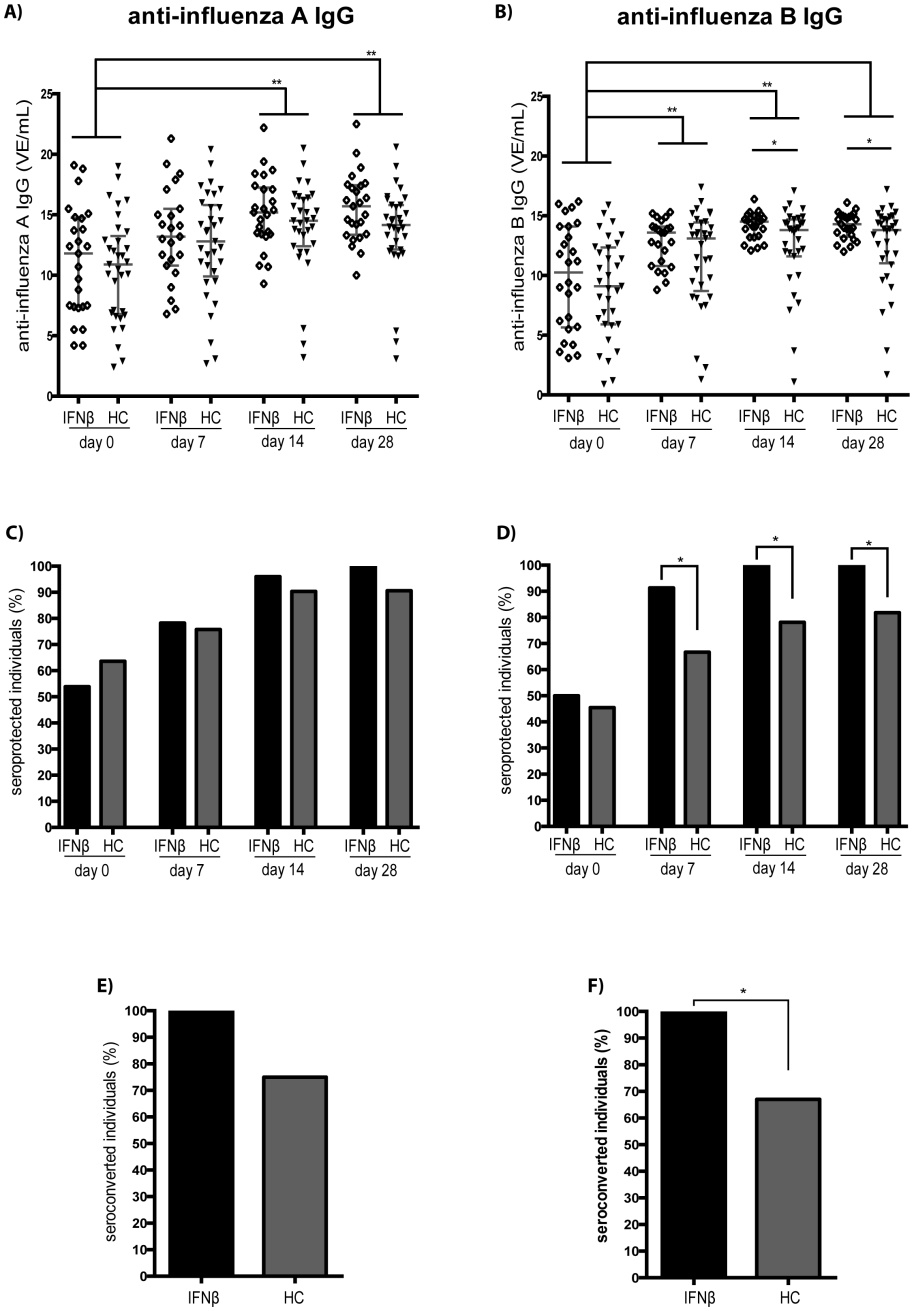
Anti-influenza IgG-response after influenza-vaccination in IFNβ-treated patients and in healthy controls. The concentration of anti-influenza A (panel A) and anti-influenza B (panel B) IgG is shown as detected before (day 0) and at day 7, 14 and 28 after influenza vaccination in IFNβ-treated patients with MS (IFNβ) and healthy controls (HC) (red lines indicate the median ± IQR). The percentage of patients fulfilling IgG sero-protection criteria for influenza A (panel C) and influenza B (panel D) is shown before (day 0) and at day 7, 14 and 28 after influenza vaccination in IFNβ-treated patients with MS (IFNβ) and healthy controls (HC). The percentage of IFNβ-treated patients with MS (IFNβ), and healthy controls (HC), converting from sero-negative pre-vaccination to seroprotection following vaccination is shown for influenza A (panel E) and influenza B (panel F) (day 7–28). * indicates p< 0.05; ** indicates p< 0.001

Baseline IgG-levels specific for influenza A and B also were comparable in IFNβ-treated patients and in HC. Influenza A-specific IgG increased significantly and comparably in both study groups at day 14, and remained increased at comparable levels on day 28 post vaccination (**Fig. 2A**). Levels of anti-influenza B-specific IgG also increased significantly by day 7, and remained increased on day 14 and 28 post vaccination in both study groups. IFNβ-treated patients mounted a more pronounced response, resulting in significantly higher levels of anti-influenza B IgG on both day 14 and day 28 post vaccination (**Fig. 2B**).

Before vaccination, a respective 54% and 64% of IFNβ-treated patients and HC fulfilled predefined seroprotection criteria (IgG ≥10 VE/mL) for influenza A (p = 0.89), 50% and 46% for influenza B (p = 0.89) –indicating previous contact with antigen from these viruses in a substantial proportion of study participants (**Fig. 2C**/**D**).

At day 7 after vaccination, the proportion of individuals fulfilling seroprotection criteria towards influenza A was increased comparably in both IFNβ-treated patients and in HC. By contrast, more IFNβ-treated individuals fulfilled seroprotection criteria for anti-influenza B at this time point (p = 0.02). At days 14 and 28, 100% of the IFNβ-treated patients fulfilled seroprotection criteria for both anti-influenza A and anti-influenza B. In HC, by contrast, on day 14 and 28 seroprotection criteria for anti-influenza A were only met by 90% and 91%, for anti-influenza B by 78% and 82% (p = 0.01 for both comparisons), respectively. Also, only a respective 75% and 67% of the HC with undetectable levels of pre-vaccination IgG against influenza A and B converted to protective antibody levels –compared to 100% in IFNβ-treated patients (p = 0.03 for anti-influenza B IgG) (**Fig. 2E**/**F**). In patients with MS, no differences in vaccine-induced humoral immune responses were noted between the used IFNβ-preparations.

### Cellular vaccine-specific immune response

**Figure 3:**
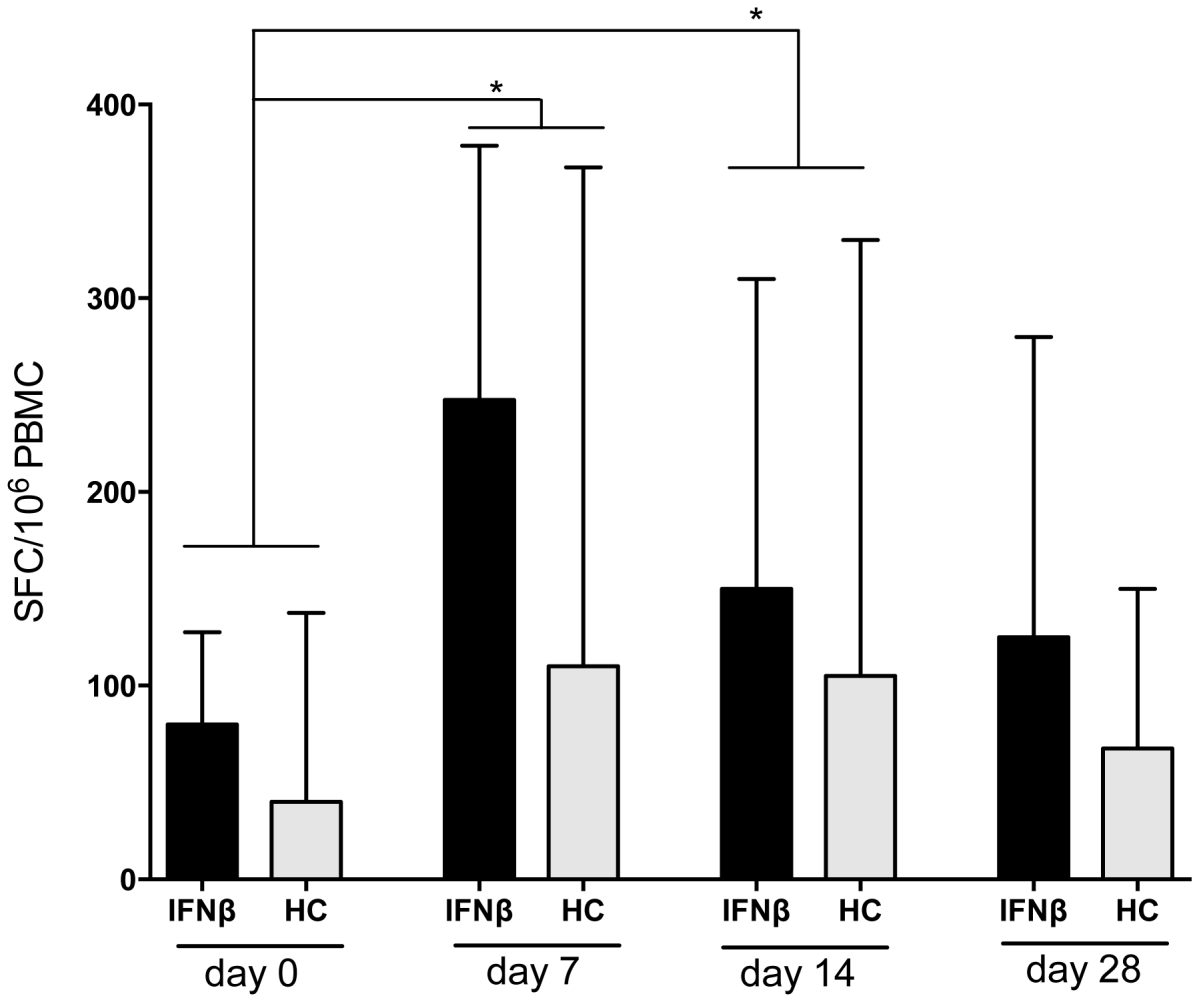
Cellular immune response after influenza-vaccination in IFNβ-treated patients vs. healthy controls. The frequency of influenza-specific cells in IFNβ-treated patients with MS (IFNβ and healthy controls (HC) as detected by spot forming cells (SFC) in equal amounts of peripheral blood mononuclear cells (PBMC) is shown before (day 0) and at day 7, 14 and 28 after influenza vaccination (median + IQR). * indicates p<0.05.

The frequency of T cells producing IFN-gamma in response to influenza-antigen was assessed by ELISpot. Before vaccination, frequencies of influenza-specific IFN-gamma secreting T cells were comparable in IFNβ-treated patients and in HC, as was the number of individuals with no detectable influenza-specific cellular response. By day 7 post-vaccination, frequencies significantly increased in both groups and reached similar levels (HD: p =  0.0093; MS- IFNβ: p = 0.025)(**Fig. 3**). Numbers of influenza-specific T cells remained similarly increased until day 14 post-vaccination in both study groups. By day 28 post-vaccination, frequencies of IFN-gamma-secreting cells contracted to pre-vaccination levels in both groups. The proportion of patients with a strong vaccine-specific cellular immune response (predefined cut-off: >250 SFC/10^6^ PBMC) was also comparable in both groups at all post-vaccination time-points (data not shown). Of note, at all time points a tendency towards a higher frequency of vaccine-specific T cells in IFNβ-treated patients was evident when compared to controls. Again, in patients with MS no differences in the vaccine-induced cellular immune response were noted between the used IFNβ-preparations.

## Discussion

The key observation of our study was that in individuals responding clinically and radiologically to treatment with IFNβ vaccine-specific humoral and cellular immune responses are preserved.

Also, previous studies assessing vaccine-specific immune responses in patients treated with IFNβ have shown no differences when compared to healthy controls or untreated patients with MS [11], [12], [14]. However, these studies have not been stratified with regard to therapeutic response to IFNβ. Depending on the criteria for therapeutic response being used, up to 47% of the patients have been reported not to respond to treatment with IFNβ [15], [16]. Therefore, the above-mentioned vaccination-studies might have missed immunological effects in patients responding to IFNβ-therapy. However, in comparison to controls we found in our cohort with documented reduction of the relapse rate and the number of new T2-lesions in MRI, preserved vaccine specific immune responses. This finding does not support subclinical immune-inhibitory effects of IFNβ. By contrast, our data indicate that antigen-specific immune responses in IFNβ-treated patients with MS are at least comparable to controls.

Besides possibly uniform immunological activity of IFNβ, also pleiotropic effects of the cytokine have been discussed [17]–[21]. In our study the proportion of patients with general symptoms following vaccination, vaccination-induced influenza B seroprotection, and the rate of conversion from undetectable to protective anti-influenza B IgG levels was higher in IFNβ-treated individuals. However, neither was vaccine-induced humoral immune response consistently increased, nor was the vaccine-specific cellular immune response enhanced. Therefore, a general pleiotropic effect cannot be derived from our data.

Limitations of our study are (i) the lack of a control group of MS-patients that do not respond to treatment with IFNβ, missing information on correlations of the vaccine-response with (ii) a potential IFNβ-induced lymphopenia and (iii) the MHC haplotype status of our study subjects. However, the broadened therapeutic options for MS patients that do not respond to first-line therapies prevented us from recruiting patients with continuous inflammatory disease activity under therapy with IFNβ. Additional limitations of our study are its insufficient power to evaluate clinical endpoints (such as protection from influenza infection), the retrospective nature of the MS-disease activity assessment, and that an –albeit unlikely– MS-intrinsic effect, that has been indicated in yet small studies [17], [22], cannot be ultimately excluded. However, comprehensively investigating for the first time in a cohort of patients with MS the clinical and radiologic course of disease as well as both humoral and cellular vaccine-specific immune responses, our data indicate preserved antigen-specific immunity in IFNβ-treated individuals. For clinicians, knowledge of this can be informative when discussing with IFNβ-treated patients questions related to vaccinations.
